# Preoperative Anxiety Before Paediatric Dental Rehabilitation Under General Anaesthesia: Associations with Accompanying Parent Characteristics and Caries Experience

**DOI:** 10.3390/children13101319

**Published:** 2026-09-28

**Authors:** Meltem Karahan, Zeynep Solmazgul Yazici, Basak Kiziltan Eliacik, Kursad Nuri Baydili

**Affiliations:** 1 Department of Pediatric Dentistry, Hamidiye Faculty of Dental Medicine, University of Health Sciences, 34668 Istanbul, Türkiye; zeynep.solmazgulyazici@sbu.edu.tr (Z.S.Y.); basak.eliacik@sbu.edu.tr (B.K.E.); 2 Department of Biostatistics and Medical Informatics, Hamidiye Faculty of Medicine, University of Health Sciences, 34668 Istanbul, Türkiye; kursadnuri.baydili@sbu.edu.tr

**Keywords:** general anesthesia, anxiety, child, dental caries, parents, pediatric dentistry

## Abstract

**Highlights:**

**What are the main findings?**

- Child preoperative anxiety was not correlated with parental state or trait anxiety, and caries experience was not associated with child or parental anxiety.
- Higher child anxiety was observed among children accompanied by fathers and by parents older than 35 years; fathers also reported higher state anxiety than mothers.

**What are the implications of the main findings?**

- Preoperative assessment may benefit from considering characteristics of the accompanying parent alongside the child’s anxiety level.
- Standardized, family-centered assessment and preparation may help address the individual needs of each child–caregiver.

**Abstract:**

**Background/Objectives:** Preoperative anxiety may complicate the delivery of comprehensive dental rehabilitation under general anaesthesia, yet evidence regarding child–parent anxiety relationships and dental disease burden in paediatric dental settings is limited. To evaluate children’s preoperative anxiety and its associations with accompanying parents’ state and trait anxiety, parent characteristics and dental caries experience. **Methods:** This cross-sectional study was conducted in a tertiary dental hospital in Istanbul, Türkiye, and included 61 children aged 3–9 years with ASA physical status I–II who were undergoing dental rehabilitation under general anaesthesia and their accompanying parents. Child anxiety was assessed immediately before transfer to the operating room using the Modified Yale Preoperative Anxiety Scale; parental anxiety was assessed using the State–Trait Anxiety Inventory. Caries experience was recorded using dmft/DMFT indices. Non-parametric group comparisons and Spearman correlation analyses were performed. **Results:** The mean child anxiety score was 51.37 ± 21.08. Children accompanied by fathers had higher scores than those accompanied by mothers (*p* = 0.018), and children accompanied by parents older than 35 years had higher scores than those accompanied by younger parents (*p* = 0.037). Fathers had higher state anxiety scores than mothers (*p* = 0.008). No significant correlations were observed between child anxiety and parental state or trait anxiety, or between caries experience and child or parental anxiety (all *p* > 0.05). **Conclusions:** No significant relationship was observed between parental anxiety and children’s preoperative anxiety. However, children accompanied by fathers and parents older than 35 years had higher preoperative anxiety scores.

## 1. Introduction

Dental fear and anxiety are multifactorial phenomena that may arise from the interaction of innate temperamental characteristics and acquired environmental influences, including social learning and previous negative experiences [1]. Children may be particularly susceptible to these influences, as negative treatment-related stories shared by peers or threatening expressions used by parents can reinforce dental fear and anxiety. Children’s tendency to perceive events differently from adults complicates both managing the clinical process and implementing psychological approaches [2,3].

For children, hospitalization can be an emotionally distressing and potentially traumatic experience. In this setting, the development of anxiety and fear is commonly observed among children [4]. In pediatric dentistry, general anesthesia may be indicated when conventional behavior guidance techniques are insufficient, particularly in very young or uncooperative children and in those requiring extensive or comprehensive dental treatment [5]. Despite facilitating the delivery of necessary dental care in such cases, general anesthesia itself may represent a major source of anxiety for both children and their parents. Anxiety is defined as feelings of worry, uneasiness, and fear that arise in response to a perceived or actual threat [6,7].

Preoperative anxiety is common among children undergoing treatment under general anesthesia and may lead to adverse physiological and psychological responses [4]. Factors influencing preoperative anxiety include the child’s social adaptability, cognitive capacity, temperament, age, previous negative medical experiences, parental state anxiety level, and parental stress management [8]. Evidence from previous research suggests that younger age, certain temperamental characteristics, previous adverse medical experiences, and parent–child relational factors may contribute to an increased susceptibility to preoperative anxiety in pediatric patients [9]. Although the indication for general anesthesia is established before the day of treatment, assessing preoperative anxiety may still have clinical value. Identifying highly anxious children and parents may help clinicians tailor perioperative communication, preparation, and supportive strategies to individual needs. Such assessment may therefore contribute to a more patient- and family-centered perioperative experience, even when the planned anesthetic pathway remains unchanged [6,10].

A previous study reported that prior hospitalization of the child, being an only child, and parental anxiety levels were associated with increased preoperative anxiety in children. The same study also suggested that parental anxiety may be influenced by several factors, including the age of the child and parent, female sex, lower or higher educational attainment, residence in a rural area, and an anxiety-prone personality trait, namely trait anxiety [11].

Previous studies in pediatric dentistry have examined the relationship between parental anxiety and children’s dental anxiety, as well as the possible association between dental anxiety and caries experience [3,12]. Recent evidence also shows that children with adverse dental histories and greater caries burden may require more advanced behavior guidance approaches, including treatment under general anesthesia [13]. However, evidence specifically examining children’s preoperative anxiety together with parental anxiety and caries experience in the dental general anesthesia setting remains limited. Evaluating these factors together may therefore provide a clearer understanding of preoperative anxiety in this particular patient group.

This study aimed to evaluate preoperative anxiety levels in children with ASA physical status I–II scheduled to undergo dental treatment under general anesthesia in Türkiye, and to examine the relationship between children’s preoperative anxiety and parental anxiety levels, as well as its possible association with dental caries experience, assessed using the dmft index.

The null hypothesis (H_0_) was that children’s preoperative anxiety levels would not be significantly associated with parental anxiety levels and dental caries experience, as assessed using the dmft index. The alternative hypothesis (H_1_) was that children’s preoperative anxiety levels would be significantly associated with parental anxiety levels and dental caries experience, as assessed using the dmft index.

## 2. Materials and Methods

### 2.1. Study Design, Setting and Participants

This cross-sectional observational study was conducted in the paediatric dentistry clinic of a tertiary oral and dental health training and research hospital in Istanbul, Türkiye. The University of Health Sciences Hamidiye Non-Interventional Scientific Research Ethics Committee approved the study (12 June 2025; approval no. 13/18), and the study complied with the Declaration of Helsinki and obtained written informed consent from the parents or legal guardians of all participating children. Data collection was conducted between 14 November 2025, and 24 April 2026.

Children aged 3–9 years scheduled for dental treatment under general anaesthesia, along with their accompanying parents, were screened. Inclusion criteria were American Society of Anesthesiologists physical status I or II and an indication for dental treatment under general anaesthesia. Children with developmental, neurological or psychiatric disorders, special healthcare needs, preoperative medication related to systemic disease, or emergency surgical indications were excluded. Parents who could not read or understand Turkish, had inadequate literacy to complete the instruments, declined participation or did not provide consent were excluded. No routine preoperative premedication was administered. In accordance with the routine clinical practice of the unit, the accompanying parent was determined by family circumstances and was not assigned by the investigators.

### 2.2. Data Collection

Data were collected in the preoperative waiting area on the day of treatment. A face-to-face interview recorded child age, sex, birth order, number of children in the family and previous hospitalisation, together with the accompanying parent’s age, sex and educational level.

### 2.3. Clinical Examination

Dental caries experience was assessed during treatment under general anaesthesia according to World Health Organization criteria [14]. Examinations were performed with a dental mirror (LM-Instruments Oy, Parainen, Finland) and a World Health Organization periodontal probe (LM-Instruments Oy, Parainen, Finland). Decayed, missing and filled primary and permanent teeth were recorded, and individual dmft and DMFT scores were calculated.

### 2.4. Assessment of Child Preoperative Anxiety

Child preoperative anxiety was evaluated immediately before transfer to the operating room using the Modified Yale Preoperative Anxiety Scale (m-YPAS), an observational instrument covering activity, vocalisation, emotional expressivity, state of arousal and use of parents [15]. The validated Turkish version was used [16,17]. Higher scores indicate greater anxiety. Two trained pediatric dentists independently and simultaneously evaluated each child during the same brief preoperative observation period immediately before transfer to the operating room. The observers were unaware of each other’s ratings during the assessment. After completing their individual ratings, they left the observation area and compared their scores. When discrepancies were present, consensus was reached, and the consensus score was used in the analyses.

### 2.5. Assessment of Parental Anxiety

The accompanying parent completed the Turkish State–Trait Anxiety Inventory (STAI) in the preoperative waiting area before the child entered the operating room [18,19,20]. The state (STAI-S) and trait (STAI-T) subscales each contain 20 items and yield scores from 20 to 80, with higher scores indicating greater anxiety.

### 2.6. Statistical Analysis

The primary analysis evaluated the correlation between child m-YPAS and parental STAI scores. In the absence of a directly comparable study for sample size estimation, the calculation was based on detecting a correlation of at least r = 0.40 with 90% power at α = 0.05; 61 child–parent dyads were required (G*Power version 3.1.9.7, Heinrich Heine University Düsseldorf, Düsseldorf, Germany).

Analyses were performed using IBM SPSS Statistics, version 27.0 (IBM Corp., Armonk, NY, USA). Distributional assumptions were assessed with the Kolmogorov–Smirnov test. Quantitative variables are reported as mean ± standard deviation and median (range), and categorical variables as number and percentage. The Mann–Whitney U test was used for two-group comparisons and the Kruskal–Wallis test for comparisons involving three or more groups. Spearman rank correlation assessed relationships among m-YPAS, STAI-S, STAI-T, dmft and DMFT scores. Secondary demographic comparisons were exploratory; no adjustment for multiple testing was applied. Two-sided *p* < 0.05 was considered statistically significant.

## 3. Results

### 3.1. Participant and Clinical Characteristics

The 61 children included in the study were 3–9 years old (mean, 5.08 ± 1.13 years); 35 (57.4%) were boys. Mean dmft and DMFT scores were 12.08 ± 3.74 and 0.10 ± 0.40, respectively. The accompanying parents had a mean age of 36.11 ± 6.01 years; 41 (67.2%) were mothers and 20 (32.8%) were fathers. Participant characteristics are summarised in Table 1.

### 3.2. Anxiety Outcomes and Group Comparisons

The mean m-YPAS score was 51.37 ± 21.08. Mean parental STAI-S and STAI-T scores were 41.20 ± 5.99 and 47.00 ± 6.26, respectively.

Children accompanied by fathers had higher m-YPAS scores than children accompanied by mothers (61.17 ± 24.74 vs. 46.59 ± 17.44; *p* = 0.018). Children accompanied by parents older than 35 years also had higher m-YPAS scores than those accompanied by parents aged 35 years or younger (56.72 ± 21.62 vs. 45.83 ± 19.33; *p* = 0.037). Fathers reported higher state anxiety than mothers (43.75 ± 5.17 vs. 39.95 ± 6.03; *p* = 0.008). No significant differences in m-YPAS, STAI-S or STAI-T scores were observed according to parental educational level, child age group, child sex, birth order, number of children in the family or previous hospitalisation (Table 2).

Children accompanied by fathers had higher m-YPAS scores than children accompanied by mothers (61.17 ± 24.74 vs. 46.59 ± 17.44; *p* = 0.018, r = 0.30). Children accompanied by parents older than 35 years also had higher m-YPAS scores than those accompanied by parents aged 35 years or younger (56.72 ± 21.62 vs. 45.83 ± 19.33; *p* = 0.037, r = 0.27). Fathers reported higher state anxiety than mothers (43.75 ± 5.17 vs. 39.95 ± 6.03; *p* = 0.008, r = 0.34).

### 3.3. Correlation Analyses

Child m-YPAS scores were not correlated with parental STAI-S (r_s_ = 0.096, *p* = 0.463) or STAI-T scores (r_s_ = 0.041, *p* = 0.753). Neither dmft nor DMFT was significantly correlated with child or parental anxiety scores (all *p* > 0.05; Table 3).

## 4. Discussion

This study aimed to examine the relationship between preoperative anxiety levels in pediatric patients scheduled for dental treatment under general anesthesia and parental anxiety, sociodemographic variables, and caries experience. The study hypothesis that parental sociodemographic characteristics play a determining role in children’s preoperative anxiety levels was partially accepted, as it was statistically supported in terms of parental age and gender. However, this hypothesis was rejected for parental education level and child-related demographic characteristics, including age, sex, birth order, number of siblings, and previous hospital experience. In addition, the two other main research hypotheses were both rejected, as no statistically significant associations were identified. These hypotheses proposed that caries experience assessed during clinical examination would increase preoperative anxiety levels and that a positive correlation would exist between parents’ state/trait anxiety levels and children’s anxiety scores.

The demographic profile of patients requiring general anesthesia in pediatric dentistry appears to be in line with the findings of the present study. A study conducted in Germany reported that most children who underwent dental treatment under general anesthesia were between 1 and 5 years of age, and that the main indication for anesthesia was lack of cooperation despite the absence of systemic disease [21]. Similarly, a retrospective study conducted at a university hospital in South Korea reported that children aged 5–8 years constituted the largest age group among pediatric patients who underwent dental treatment under general anesthesia [22]. In the present study, the fact that the majority of the sample consisted of children aged 5 years and younger supports the notion that children in this developmental period may have limited ability to tolerate the treatment burden associated with dental procedures in the dental chair and may therefore require advanced pharmacological behavior management techniques more frequently.

Sociodemographic factors associated with preoperative anxiety appear to vary considerably between children and parents. A university hospital-based study on minor surgical procedures identified maternal status and parental educational level as independent predictors of parental anxiety, while previous hospitalization and being an only child were reported as factors associated with preoperative anxiety in children [11]. In contrast, the present study found no statistically significant differences in parental anxiety levels or children’s m-YPAS scores according to parental educational level, children’s previous hospitalization history, being an only child, or the number of children in the family. The most notable discrepancy was observed for parental gender. These differences may be related to sociocultural factors, the specific clinical context of the study, and variations in family structure.

Previous studies investigating the association between parental gender and preoperative anxiety have commonly reported higher anxiety levels among mothers compared with fathers. In a study published in 1996, having a child younger than one year of age and experiencing the child’s first surgical procedure were identified as significant predictors of increased maternal anxiety, while no significant effect of these factors was observed among fathers [23]. A study conducted in a children’s hospital found clinically significant anxiety in 42% of parents of children scheduled for elective surgery and demonstrated that mothers experienced higher levels of anxiety than fathers [24]. In contrast to these studies, the present study found that neither the child’s age nor previous hospitalization history was significantly associated with parental anxiety. Moreover, fathers exhibited significantly higher state anxiety levels than mothers. These findings indicate that, in the present sample, paternal accompaniment was associated with both higher parental state anxiety and higher child preoperative anxiety. However, given the cross-sectional design, these findings should not be interpreted as indicating a causal relationship between paternal anxiety and children’s preoperative anxiety. The lack of an observed effect of child age on parental anxiety may be related to differences in the age distribution of the sample compared with previous studies. More importantly, considering the time elapsed since the study by Litman et al. [23], differences between the present findings and earlier studies may also reflect changes in family roles and the increasing involvement of fathers in children’s medical and dental care; however, this interpretation remains speculative and requires further investigation.

The potential influence of parental presence on child psychology remains controversial not only in terms of which parent accompanies the child, but also regarding the number of accompanying parents. A recent study compared the presence of a single parent selected by children undergoing elective surgery with the presence of both parents during the perioperative period. The study reported that children accompanied by both parents had significantly lower anxiety scores both in the waiting area and during anesthesia induction [25]. In the current clinical setting, where children are accompanied by only one parent, children accompanied by their fathers had significantly higher m-YPAS scores, and fathers had significantly higher state anxiety scores compared with sessions in which mothers were present. When considered together with previous findings on dual-parent presence, these results suggest that the role and composition of parental accompaniment may warrant further investigation in pediatric dental general anesthesia settings.

The psychological interaction between parents and children is not limited to general medical or surgical operating room settings but has also been frequently reported in dental clinical environments. Previous research has shown that age may be associated with anxiety levels in children, whereas gender and dmft/DMFT scores, which reflect caries burden and treatment history, have not shown significant associations with anxiety [26]. The findings of the present study are consistent with these results. No statistically significant correlation was found between children’s caries burden, expressed as dmft and DMFT scores, and children’s m-YPAS anxiety scores or parents’ state and trait anxiety scores. These findings suggest that preoperative anxiety and fear experienced by children may be influenced more by immediate and situational factors than by objective indicators of previous clinical disease or treatment burden.

A study conducted in Italy examining parental anxiety and its associated factors during children’s surgical procedures reported that disclosure of surgical complications increased parental anxiety, whereas providing practical information, such as pain management strategies, helped reduce anxiety [27]. More recently, another study demonstrated that non-pharmacological visual and auditory information provided to parents through storybooks or videos during the preoperative period significantly reduced parental state anxiety compared with the control group [28]. Accordingly, structured perioperative communication and systematic information provision may be valuable components of strategies aimed at addressing preoperative anxiety. In addition, incorporating customized visual educational materials that explain pediatric dental procedures into routine clinical practice may indirectly enhance children’s clinical comfort.

This study has several limitations. The limited sample size and single-center design may restrict the validity and generalizability of the results to broader pediatric populations. In addition, anxiety was evaluated only during the acute preoperative period; therefore, postoperative anxiety responses and emergence agitation were not assessed. The accompanying parent was determined by family circumstances rather than being assigned by the investigators. Therefore, self-selection bias cannot be excluded, as the parent who accompanied the child may have differed systematically in anxiety level from the parent who did not attend. This should be considered when interpreting the findings related to parental gender. Additional psychosocial and family-structure variables, such as parental loss, marital status, parental separation or divorce, and intra-familial dynamics, were not assessed. These unmeasured factors may have influenced both child and parental anxiety and therefore represent potential residual confounding. Although the two observers performed their assessments independently and simultaneously to minimise variability related to changes in the child’s preoperative anxiety over time, the individual pre-consensus ratings were not retained separately. Therefore, inter-rater reliability could not be quantified retrospectively, which should be considered a methodological limitation. Finally, because multiple secondary demographic comparisons were performed without adjustment for multiple testing, the significant findings related to parental age and parental gender should be interpreted cautiously, as the possibility of Type I error cannot be excluded. Future studies with larger, multicenter samples are needed to validate these findings. Moreover, research designs incorporating both quantitative and qualitative methods may provide a more comprehensive understanding of the preoperative and postoperative anxiety experiences of both mothers and fathers. Such studies may contribute to addressing the existing gap in the literature regarding parental factors associated with perioperative anxiety in children undergoing dental treatment under general anesthesia.

## 5. Conclusions

Child preoperative anxiety before dental rehabilitation under general anaesthesia was not correlated with accompanying parents’ state or trait anxiety and was not associated with caries experience. Higher anxiety scores among children accompanied by fathers or by parents older than 35 years were exploratory findings in a setting where parent accompaniment was not assigned. Standardised, family-centred assessment and preparation should therefore address the needs of each child–caregiver dyad rather than rely on parent-specific assumptions.

## Figures and Tables

**Table 1:** Demographic and clinical characteristics of participating children and accompanying parents (*n* = 61).

Characteristic	Category	n (%)/Mean ± SD	Median (Range)
**Accompanying parent characteristics**			
Age, years	—	36.11 ± 6.01	36 (25–49)
Age group	≤35 years	30 (49.2)	—
	>35 years	31 (50.8)	—
Accompanying parent	Mother	41 (67.2)	—
	Father	20 (32.8)	—
Educational level	Primary school	10 (16.4)	—
	Middle school	20 (32.8)	—
	High school	17 (27.9)	—
	University or higher	14 (22.9)	—
**Child characteristics**			
Age, years	—	5.08 ± 1.13	5 (3–9)
Age group	≤5 years	42 (68.9)	—
	>5 years	19 (31.1)	—
Sex	Female	26 (42.6)	—
	Male	35 (57.4)	—
Birth order	First child	19 (31.1)	—
	Second child	18 (29.5)	—
	Third or later	24 (39.3)	—
Children in family	One child	11 (18.0)	—
	Two children	21 (34.4)	—
	Three or more children	29 (47.5)	—
Previous hospitalisation	No	50 (82.0)	—
	Yes	11 (18.0)	—
dmft index	—	12.08 ± 3.74	12 (3–20)
DMFT index	—	0.10 ± 0.40	0 (0–2)

Values are *n* (%) unless otherwise indicated. SD, standard deviation; dmft, decayed, missing and filled primary teeth; DMFT, decayed, missing and filled permanent teeth.

**Table 2:** Child preoperative anxiety and parental anxiety scores according to sociodemographic characteristics.

Variable	Category	*n*	m-YPAS		STAI-S		STAI-T	
			Mean ± SD	*p*	Mean ± SD	*p*	Mean ± SD	*p*
Parental age	≤35 years	30	45.83 ± 19.33	**0.037 (r = 0.27)**	41.17 ± 5.99	0.701	46.93 ± 4.96	0.654
	>35 years	31	56.72 ± 21.62		41.23 ± 6.10		47.06 ± 7.39	
Accompanying parent	Mother	41	46.59 ± 17.44	**0.018 (r = 0.30)**	39.95 ± 6.03	**0.008 (r = 0.34)**	47.05 ± 6.91	0.683
	Father	20	61.17 ± 24.74		43.75 ± 5.17		46.90 ± 4.82	
Parental educational level	Primary school	10	42.00 ± 16.48	0.096	40.10 ± 5.95	0.816	49.60 ± 10.83	0.957
	Middle school	20	50.33 ± 18.26		40.90 ± 7.09		46.90 ± 6.13	
	High school	17	49.22 ± 25.35		42.35 ± 6.30		46.29 ± 3.62	
	University or higher	14	62.14 ± 19.59		41.00 ± 4.02		46.14 ± 4.70	
Child age group	≤5 years	42	52.18 ± 21.25	0.628	41.31 ± 5.59	0.797	47.45 ± 5.50	0.164
	>5 years	19	49.56 ± 21.16		40.95 ± 6.97		46.00 ± 7.77	
Child sex	Female	26	51.60 ± 23.04	0.965	40.54 ± 4.97	0.549	46.27 ± 5.15	0.544
	Male	35	51.19 ± 19.84		41.69 ± 6.68		47.54 ± 7.00	
Birth order	First child	19	56.14 ± 21.14	0.280	41.42 ± 6.89	0.081	47.16 ± 4.17	0.858
	Second child	18	53.06 ± 19.68		38.94 ± 3.33		46.28 ± 5.37	
	Third or later	24	46.32 ± 21.80		42.71 ± 6.48		47.42 ± 8.18	
Children in family	One child	11	55.30 ± 15.84	0.471	40.55 ± 7.65	0.491	47.36 ± 5.01	0.924
	Two children	21	54.05 ± 23.66		40.57 ± 4.59		46.38 ± 4.08	
	Three or more children	29	47.93 ± 20.95		41.90 ± 6.33		47.31 ± 7.92	
Previous hospitalisation	No	50	52.03 ± 21.63	0.651	41.68 ± 5.76	0.292	47.44 ± 6.27	0.271
	Yes	11	48.33 ± 18.97		39.00 ± 6.83		45.00 ± 6.13	

Values are mean ± SD. Mann–Whitney U tests were used for two-group comparisons and Kruskal–Wallis tests for comparisons of three or more groups. Bold *p* values indicate statistical significance (*p* < 0.05). m-YPAS, Modified Yale Preoperative Anxiety Scale; STAI-S, State Anxiety subscale; STAI-T, Trait Anxiety subscale. Effect size r was calculated as |Z|/√N and reported for statistically significant Mann–Whitney U comparisons. Values of 0.10, 0.30 and 0.50 were interpreted as small, medium and large effects, respectively.

**Table 3:** Spearman correlations between dental caries indices, child preoperative anxiety and parental anxiety.

Variable	m-YPAS		STAI-S		STAI-T	
	r_s_	*p*	r_s_	*p*	r_s_	*p*
dmft	−0.134	0.302	−0.037	0.778	−0.062	0.634
DMFT	−0.028	0.829	0.131	0.313	−0.003	0.983
m-YPAS	—	—	0.096	0.463	0.041	0.753

r_s_, Spearman rank correlation coefficient; dmft, decayed, missing and filled primary teeth; DMFT, decayed, missing and filled permanent teeth; m-YPAS, Modified Yale Preoperative Anxiety Scale; STAI-S, State Anxiety subscale; STAI-T, Trait Anxiety subscale.

## Data Availability

De-identified data are available from the corresponding author upon reasonable request. Original paper forms are retained securely by the research team.

## References

[1] Piechal A., Siekierska E., Blecharz-Klin K. (2026). Etiology of dental anxiety and dental phobia: Review. Eur. J. Dent..

[2] Busato P., Garbín R.R., Santos C.N., Paranhos L.R., Rigo L. (2017). Influence of maternal anxiety on child anxiety during dental care: Cross-sectional study. Sao Paulo Med. J..

[3] Bayón G., Stiernhufvud F., Ribas-Pérez D., Biedma Perea M., Mendoza Mendoza A. (2025). Parental anxiety disorders and their impact on dental treatment in children aged 4 to 13 years: A cross-sectional observational study. J. Clin. Med..

[4] Mustafa M.S., Shafique M.A., Zaidi S.D.E.Z., Qamber A., Rangwala B.S., Ahmed A., Zaidi S.M.F., Rangwala H.S., Uddin M.M.N., Ali M. (2024). Preoperative anxiety management in pediatric patients: A systemic review and meta-analysis of randomized controlled trials on the efficacy of distraction techniques. Front. Pediatr..

[5] American Academy of Pediatric Dentistry (2025). Behavior guidance for the pediatric dental patient. The Reference Manual of Pediatric Dentistry.

[6] Aimaiti Y., Wang Y., Lai A., Yang Y., Zhou Z., Li Y., Qiu Y., Shen X. (2026). Parental anxiety and preoperative anxiety in children: A dose–response association in the perioperative setting. J. Perianesth. Nurs..

[7] Cagiran E., Sergin D., Deniz M.N., Tanattı B., Emiroglu N., Alper I. (2014). Effects of sociodemographic factors and maternal anxiety on preoperative anxiety in children. J. Int. Med. Res..

[8] Liu W., Xu R., Jia J., Shen Y., Li W., Bo L. (2022). Research progress on risk factors of preoperative anxiety in children: A scoping review. Int. J. Environ. Res. Public Health.

[9] Ahmed M.I., Farrell M.A., Parrish K., Karla A. (2011). Preoperative anxiety in children: Risk factors and non-pharmacological management. Middle East J. Anaesthesiol..

[10] Bataineh M., Al-Skafi A.A., AlJundi S.H. (2025). The effect of a preoperative preparation video on preoperative anxiety among children undergoing dental treatment under general anesthesia: A randomized controlled trial. Int. J. Paediatr. Dent..

[11] Charana A., Tripsianis G., Matziou V., Vaos G., Iatrou C., Chloropoulou P. (2018). Preoperative anxiety in Greek children and their parents when presenting for routine surgery. Anesthesiol. Res. Pract..

[12] Esian D., Bica C., Vlasa A., Bud E., Stepco E., Bud A., Beresescu L. (2024). Retrospective study regarding the correlation between dental anxiety and color preferences in children with severe early childhood caries. Dent. J..

[13] Hashemi F., Al Masri A., Splieth C.H., Schmoeckel J. (2025). Clinical pathways in managing high-caries children with adverse dental histories. Medicina.

[14] World Health Organization (2013). Oral Health Surveys: Basic Methods.

[15] Kain Z.N., Mayes L.C., Cicchetti D.V., Bagnall A.L., Finley J.D., Hofstadter M.B. (1997). The Yale Preoperative Anxiety Scale: How does it compare with a “gold standard”?. Anesth. Analg..

[16] Hatipoğlu Z., Kırdök O., Özcengiz D. (2019). Validity and reliability of the Turkish version of the modified Yale Preoperative Anxiety Scale. Turk. J. Med. Sci..

[17] Topalel S., Orekici Temel G., Azizoglu M. (2020). Evaluation of preoperative anxiety in Turkish paediatric patients and validity and reliability of the Turkish Modified Yale Preoperative Anxiety Scale. Turk. J. Anaesthesiol. Reanim..

[18] Spielberger C.D. (1983). Manual for the State-Trait Anxiety Inventory STAI (Form Y).

[19] Spielberger C.D., Gorsuch R.L., Lushene R.E. (1970). Manual for the State-Trait Anxiety Inventory (Self-Evaluation Questionnaire).

[20] Öner N., LeCompte W.A. (1983). Durumluk-Sürekli Kaygı Envanteri El Kitabı.

[21] Kries T., Rupf S., Hannig M., Naim J., Gund M.P. (2023). The impact of general medical health status, demographical, and patient-specific variables on need for dental treatment of children and adolescents under general anesthesia. Clin. Oral Investig..

[22] Shin B., Yoo S., Kim J., Kim S., Kim J. (2016). A survey of dental treatment under general anesthesia in a Korean university hospital pediatric dental clinic. J. Dent. Anesth. Pain Med..

[23] Litman R.S., Berger A.A., Chhibber A. (1996). An evaluation of preoperative anxiety in a population of parents of infants and children undergoing ambulatory surgery. Pediatr. Anesth..

[24] Cameron J.A., Bond M.J., Pointer S.C. (1996). Reducing the anxiety of children undergoing surgery: Parental presence during anaesthetic induction. J. Paediatr. Child Health.

[25] Yarımoglu R., Sezgin A., Duran A., Yucedag F., Basaran B. (2026). The impact of the presence of both parents on perioperative anxiety in children undergoing adenotonsillectomy surgery: A randomized clinical trial. Acta Anaesthesiol. Scand..

[26] Jervøe-Storm P.-M., Peters L.P., Bekes K., Fricke M., Jepsen S. (2023). Evaluation of children’s anxiety level in relation to a dental visit/treatment and their parents’ dental fear. J. Clin. Med..

[27] Pomicino L., Maccacari E., Buchini S. (2018). Levels of anxiety in parents in the 24 h before and after their child’s surgery: A descriptive study. J. Clin. Nurs..

[28] Díaz Luengo T., Rivas A.B., Loureido E., Vargas E. (2023). Reducing preoperative anxiety in parents of surgical patients. Heliyon.

